# Sleep Spindles Predict Stress-Related Increases in Sleep Disturbances

**DOI:** 10.3389/fnhum.2015.00068

**Published:** 2015-02-10

**Authors:** Thien Thanh Dang-Vu, Ali Salimi, Soufiane Boucetta, Kerstin Wenzel, Jordan O’Byrne, Marie Brandewinder, Christian Berthomier, Jean-Philippe Gouin

**Affiliations:** ^1^Department of Exercise Science, Concordia University, Montréal, QC, Canada; ^2^Center for Studies in Behavioral Neurobiology, Concordia University, Montréal, QC, Canada; ^3^PERFORM Center, Concordia University, Montréal, QC, Canada; ^4^Centre de Recherche de l’Institut Universitaire de Gériatrie de Montréal, Montréal, QC, Canada; ^5^Center for Clinical Research in Health, Concordia University, Montréal, QC, Canada; ^6^Department of Psychology, Concordia University, Montréal, QC, Canada; ^7^Physip SA, Paris, France

**Keywords:** spindles, sleep, insomnia, stress, EEG

## Abstract

**Background and Aim:** Predisposing factors place certain individuals at higher risk for insomnia, especially in the presence of precipitating conditions such as stressful life events. Sleep spindles have been shown to play an important role in the preservation of sleep continuity. Lower spindle density might thus constitute an objective predisposing factor for sleep reactivity to stress. The aim of this study was therefore to evaluate the relationship between baseline sleep spindle density and the prospective change in insomnia symptoms in response to a standardized academic stressor.

**Methods:** Twelve healthy students had a polysomnography recording during a period of lower stress at the beginning of the academic semester, along with an assessment of insomnia complaints using the insomnia severity index (ISI). They completed a second ISI assessment at the end of the semester, a period coinciding with the week prior to final examinations and thus higher stress. Spindle density, amplitude, duration, and frequency, as well as sigma power were computed from C4–O2 electroencephalography derivation during stages N2–N3 of non-rapid-eye-movement (NREM) sleep, across the whole night and for each NREM sleep period. To test for the relationship between spindle density and changes in insomnia symptoms in response to academic stress, spindle measurements at baseline were correlated with changes in ISI across the academic semester.

**Results:** Spindle density (as well as spindle amplitude and sigma power), particularly during the first NREM sleep period, negatively correlated with changes in ISI (*p* < 0.05).

**Conclusion:** Lower spindle activity, especially at the beginning of the night, prospectively predicted larger increases in insomnia symptoms in response to stress. This result indicates that individual differences in sleep spindle activity contribute to the differential vulnerability to sleep disturbances in the face of precipitating factors.

## Introduction

The natural history of insomnia is hypothesized to involve three categories of factors: predisposing factors placing certain individuals at higher risk for insomnia complaints, precipitating factors triggering the onset of insomnia, and perpetuating factors maintaining the insomnia over time (Spielman, 1986). The characterization of predisposing and precipitating factors is of prime importance not only to understand the pathophysiology of insomnia but also to implement optimal preventive sleep interventions. In terms of predisposing factors, longitudinal studies have shown that the rate of new onset insomnia is higher among individuals with depressive or anxiety symptoms, a family history of insomnia, high arousability predisposition, poor general health condition, and pain syndrome (LeBlanc et al., 2009; Harvey et al., 2014). On the other hand, precipitating factors have been shown to vary with age; in particular, for younger individuals (<30 years old), stress-related factors at work/school constitute the most frequent precipitating events triggering insomnia onset (Bastien et al., 2004). Given one’s profile of predisposing factors, individuals are not equally vulnerable to the development of sleep disturbances in the face of common precipitating factors such as stressful events (Drake et al., 2004).

Beyond medical and psychological history, there has been no investigation of the inter-individual variations in sleep architecture – and sleep oscillations – as predisposing factors for the insomnia symptoms. Among the different components of sleep architecture, sleep spindles have been the subjects of intense research over the past decade (De Gennaro and Ferrara, 2003; Fogel and Smith, 2011). Spindles are defined as waxing-and-waning electroencephalography (EEG) waves oscillating at a frequency of 11–16 Hz and predominant over central EEG derivations; spindles characterize stage N2 of non-rapid-eye-movement (NREM) sleep but can also be found during stage N3 (De Gennaro et al., 2000; Iber et al., 2007). Animal and human studies converge to demonstrate that sleep spindles are generated through the interplay between specific populations of thalamic (particularly thalamic reticular) and cortical neurons (Steriade and McCarley, 2005; Schabus et al., 2007). While the density of sleep spindles varies considerably between individuals, it has been shown that spindle density remains remarkably stable within a same individual across different nights, thus constituting an individual trait (Gaillard and Blois, 1981; De Gennaro et al., 2005). Spindles have been shown correlated with measures of intellectual ability as well as with the overnight retention of various types of memory traces, suggesting an important role for spindles in brain plasticity and sleep-related memory consolidation (Gais et al., 2002; Schabus et al., 2004, 2006; Morin et al., 2008; Fogel and Smith, 2011). EEG and functional neuroimaging studies have also demonstrated that the cortical transmission of external – particularly acoustic – stimulation during sleep is drastically diminished during sleep spindles (Cote et al., 2000; Dang-Vu et al., 2011). These findings indicate that spindles isolate the cortex from the environment during sleep, contributing to the preservation of sleep stability. It was then inferred that spindle density, as a trait, might constitute a biomarker of sleep stability in the face of noise. This was confirmed by a study showing that individuals with lower spindle density were more vulnerable to sleep disruption from sounds presented throughout the night (Dang-Vu et al., 2010). Because spindles constitute an index of sleep stability, individuals with reduced spindle density might be more vulnerable to develop insomnia complaints, particularly when confronted to triggering factors such as stress. To the best of our knowledge, no study has investigated the role of sleep spindle as predisposing factor to insomnia onset.

The aim of this study was to prospectively assess whether spindle density would predict the worsening of sleep disturbances in response to a standardized stressor. We chose to follow a population of undergraduate university students during a period of increasing academic stress. In this context, assessing students at the beginning of the semester, corresponding to a lower stress period, and reevaluating them during a follow-up in the week preceding the final examinations, a period of higher stress, provides a unique opportunity to examine individual differences in the evolution of insomnia symptoms in response to a standardized stressor. The validity of this model is supported by data showing an increase of sleep disturbances in response to increased academic stress (Jernelov et al., 2009; Lund et al., 2010). Here, we hypothesized that a lower spindle density at baseline during the low stress period would prospectively predict larger increases in sleep complaints from the low to the high stress periods, therefore defining a neurophysiological vulnerability factor predisposing to the increase of insomnia symptoms.

## Materials and Methods

### Participants

This study is part of a larger project investigating the psychophysiological predictors of stress-induced sleep disturbances. Participants were young healthy students enrolled in full-time undergraduate programs in Psychology or Exercise Science at Concordia University. They were recruited at the beginning of the winter semester through local advertisements posted on the campus. Potential participants were screened using a semi-structured interview for the absence of exclusion criteria, i.e., current insomnia syndrome (APA, 2013), acute or chronic medical condition including psychiatric and sleep disorders, current use of prescribed medication (other than oral contraceptives), current use of over-the-counter sleep medication, cigarette smoking, age >30 years old, working on night shifts. Those deemed eligible then had a baseline assessment during the lower stress period, i.e., within the first 4 weeks of the 15-week academic winter semester of 2014 (January). This baseline evaluation included a self-reported assessment of sleep disturbances using the insomnia severity index (ISI), which is a 7-item questionnaire assessing the nature, severity, and impact of insomnia symptoms over the past month (Bastien et al., 2001), and the Pittsburgh sleep quality index (PSQI), which is a 19-item questionnaire assessing subjective sleep quality in the past month (Buysse et al., 1989). The baseline evaluation also involved an overnight in-lab sleep recording with polysomnography (PSG), in order to confirm the absence of sleep disorders (e.g., sleep apnea) as well as to detect sleep spindles and quantify their parameters (e.g., density). Besides sleep, a self-reported evaluation of psychological distress was obtained using the depression anxiety stress scales (DASS), which includes depression (DASS-D), anxiety (DASS-A), and stress (DASS-S) subscales (Lovibond and Lovibond, 1995). Furthermore, participants also completed at baseline the Ford insomnia response to stress test (FIRST), a questionnaire developed to assess trait sleep reactivity to stress (Drake et al., 2004). All eligible participants then had a second ISI, PSQI, and DASS assessment during the higher stress period, i.e., in the week prior to the final examination period. Participants signed an informed consent form before entering the study, which was approved by Concordia University Human Research Ethics Committee.

### PSG recording and sleep spindle analysis

Overnight PSG recordings were conducted at the PERFORM Center Sleep Laboratory, using 34-channel systems (Embla Titanium, Natus Medical, San Carlos, CA, USA) with EEG referenced to linked-mastoids (bandpass filter 0.3–100 Hz, sampling rate 256 Hz), electrooculography (EOG), electromyography (EMG; submental), nasal–oral thermocouple airflow, and transcutaneous finger pulse oximeter. Participants arrived to the sleep laboratory at least 1 h before their habitual bedtime, in order to allow sufficient time for the PSG setup. They were asked to refrain from alcohol and caffeine consumption and avoid strenuous physical exercise for at least 8 h prior to the PSG recording. Participants went to bed at their habitual bedtime (or at midnight at latest) and slept until they spontaneously woke up the next morning (or at 9 a.m. at latest). PSG was recorded and sleep stages were scored according to standard criteria (Iber et al., 2007). Sleep apnea syndrome was defined by an apnea–hypopnea index >5/h (exclusion criterion). Sleep spindles were automatically detected during stages N2 and N3 of NREM sleep on EEG C4–O2 derivation. This derivation was chosen given the well-described central predominance of sleep spindles (De Gennaro et al., 2000). The spindle detection method (Aseega software, Physip, Paris, France) used data-driven criteria in order to cope with both inter-subject and inter-recording condition variabilities (Berthomier et al., 2007). It was based on an iterative approach. The first iteration aimed at determining recording-specific thresholds, based on EEG power ratios in delta, alpha, and sigma bands. The second iteration provided precise temporal localization of the events. The final iteration enabled the validation of detected events based on frequency and duration criteria (>0.5 s). Iteration 1 and 3 dealt with raw EEG data, while iteration 2 was applied on the EEG filtered in the spindle (sigma) frequency range using frequency bands adapted to each individual based on his/her global spectral profile (median values for low and high bands were 11.9 and 15.9 Hz, respectively). The density of spindles was computed as the average number of detected spindles per 30 s EEG epoch for each subject. In addition to spindle density, other spindle parameters were also computed for each participant in order to comprehensively evaluate spindle activity: average maximum spindle amplitude (in microvolts), average spindle duration (in seconds), and average spindle frequency (in hertz). After cleaning of the main EEG artifacts, the EEG power in the adapted sigma frequency range (in squared microvolts, per 30 s epoch) was also computed using Hanning window. Spindle parameters and sigma power were first calculated considering the N2–N3 NREM sleep of the entire night. In addition, in order to take into account the variation of spindle activity across successive NREM sleep periods (De Gennaro et al., 2000), spindle parameters, and sigma power were also calculated for each NREM sleep period. These periods were defined according to standard criteria: a NREM sleep period was defined by a period of at least 15 min of NREM sleep followed by at least 5 min of REM sleep, and the first four NREM sleep periods were considered given that there are usually four NREM sleep periods during overnight sleep in adults (Feinberg and Floyd, 1979). For completeness, spindle parameters and sigma power were also calculated during stage N2 only, for the entire night as well as for each NREM sleep period, and the data from these additional analyses are presented in the supplementary material given that they showed results quite similar to those from N2–N3 combined.

### Statistical analysis

Changes in self-reported sleep quality and stress over time were first evaluated using dependent *t*-tests. The evolution of spindle parameters across NREM sleep periods was then examined using one-way repeated measures analysis of variance (ANOVA) with Bonferroni *post hoc* tests. In order to test our main hypothesis, correlations between spindle density (during N2–N3 for the whole night and for each NREM sleep period) and changes in self-reported sleep quality (score during high stress period minus score during low stress period) were calculated using Pearson product-moment correlation. Secondary analyses likewise tested the Pearson correlations between other spindle parameters (spindle amplitude, duration, frequency) or sigma power and sleep quality changes. FIRST scores were also correlated with sleep quality changes to evaluate the predictive potential of this self-reported measure of sleep reactivity to stress in the context of academic stress. All analyses were considered significant at a *p* value <0.05, and were conducted with SPSS Statistics 22.0 (IBM, New York, NY, USA).

## Results

**Figure 1:**
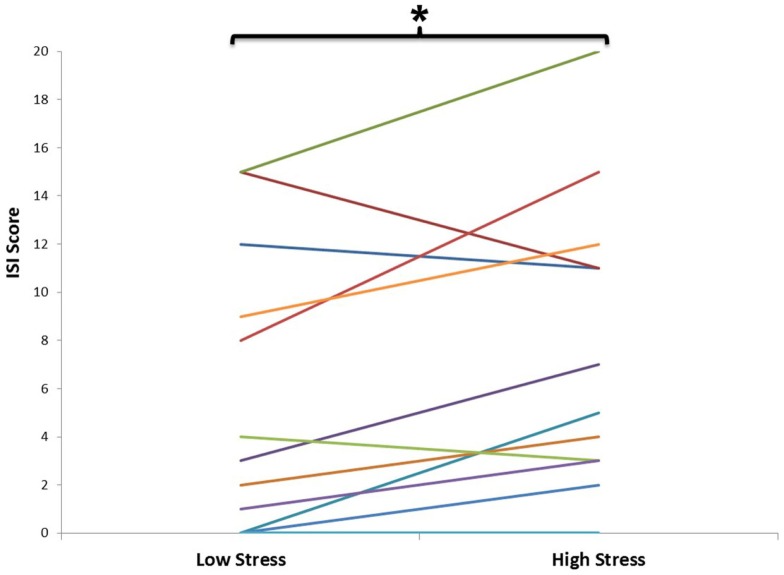
**Self-reported sleep quality, as assessed by the insomnia severity index (ISI) during low and high stress periods**. This graph depicts the evolution of ISI total score for each individual (*n* = 12) from the beginning (low stress period) to the end of the semester (high stress period). Each individual is represented by a different colored line. There was a significant increase in ISI from the low to the high stress period (*p* < 0.05).

**Figure 2:**
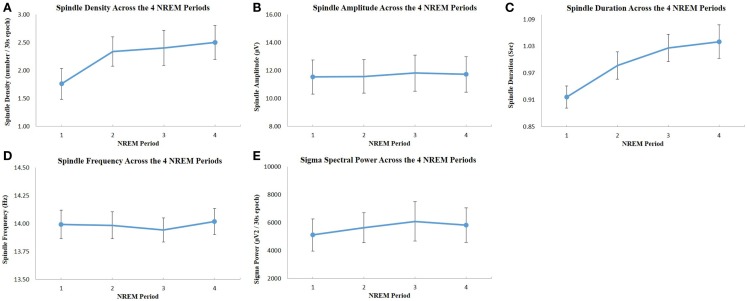
**Evolution of spindle parameters and sigma power across the four NREM sleep periods: (A) spindle density; (B) spindle maximum amplitude; (C) spindle duration; (D) spindle frequency; (E) EEG spectral power in the sigma frequency band**. All values were extracted from C4–O2 EEG derivation. The dots represent the mean value, and the bars show the standard error of the mean. There was a significant increase of spindle density and duration across NREM sleep periods (one-way repeated measures ANOVA, *p* < 0.05), but there was no significant change of spindle amplitude, frequency, and sigma power across periods.

Out of 22 potential participants, 12 were confirmed eligible and presented at least 4 NREM sleep periods during their PSG recording. The majority of them were female students (10/12). Seven of the participants were psychology majors; the remainders were exercise science majors. Characteristics of the participants, including age, PSG parameters, PSQI, and ISI values are presented in Table 1. There was a significant increase in total ISI score from the low to the high stress periods (*t* = 2.23, *p* = 0.047), as illustrated in Figure 1, but no significant change in any of the seven individual items of the ISI. There was no significant change in PSQI (*t* = 0.44, *p* = 0.67). The total DASS score significantly increased (*t* = 2.9; *p* = 0.014), including increases in depression [DASS-D: *t* = 2.55; *p* = 0.027)] and perceived stress (DASS-S: *t* = 3.48; *p* = 0.005) subscales, but not anxiety (DASS-A: *t* = 1.7; *p* = 0.12). There was a significant change in spindle density across NREM sleep periods (*F* = 4.58, *p* = 0.033); *post hoc* tests showed that spindle density during the first NREM sleep period was significantly lower than during each of the three following NREM sleep periods (*p* = 0.031, 0.025, and 0.026, respectively) (Figure 2A). Spindle duration also demonstrated a significant change across NREM sleep periods (*F* = 11.62, *p* = 0.002), with *post hoc* tests revealing a significantly lower spindle duration during NREM sleep period 1 compared to each of the next periods (*p* = 0.002) (Figure 2C). There was no significant change in spindle amplitude (*F* = 0.432, *p* = 0.735), spindle frequency (*F* = 1.664, *p* = 0.243), and sigma power (*F* = 0.869, *p* = 0.492) across NREM sleep periods (Figure 2).

**Table 1:** **Characteristics of participants (*N* = 12, 10 females)**.

Parameters	M (SD)	Range
Age	21.08 (2.43)	17–25
PSG total sleep time (min)	417.64 (62.81)	326–527.5
PSG sleep efficiency (%)	85.86 (8.81)	66.3–96.8
PSG stage N1 (% of TST)	13.01 (8.15)	3.8–30.9
PSG stage N2 (% of TST)	51.58 (6.30)	42.5–61.4
PSG stage N3 (% of TST)	18.36 (5.10)	11.8–27.5
PSG stage REM (% of TST)	17.33 (2.39)	14.1–21.3
NREM period 1 (min)	91.13 (48.10)	44–176
NREM period 2 (min)	83.83 (40.50)	30.5–175.5
NREM period 3 (min)	87.17 (37.83)	43.5–169.5
NREM period 4 (min)	61.04 (28.08)	22–123.5
Apnea-hypopnea index (nb/h)	0.31 (0.39)	0–1
Arousal index (nb/h)	8.11 (3.05)	3.3–12.5
ISI low stress	5.75 (5.82)	0–15
ISI high stress	7.75 (6.05)	0–20
PSQI low stress	5.17 (3.16)	1–10
PSQI high stress	5.42 (3.85)	0–14
DASS-D low stress	3.08 (4.56)	0–17
DASS-D high stress	4.83 (4.76)	0–17
DASS-A low stress	3 (4.24)	0–13
DASS-A high stress	4.17 (4.43)	0–15
DASS-S low stress	5.17 (4.17)	0–16
DASS-S high stress	7.92 (5.3)	0–17
DASS-total low stress	11.25 (12.39)	0–46
DASS-total high stress	16.92 (13.73)	0–49
FIRST	21.83 (7.60)	11–32

*DASS, depression anxiety stress scales (D, depression subscale; A, anxiety subscale; S, stress subscale); FIRST, Ford insomnia response to stress test; ISI, insomnia severity index; NREM period 1–4, first to fourth NREM sleep period; PSG, polysomnography; PSQI, Pittsburgh sleep quality index; TST, total sleep time*.

**Figure 3:**
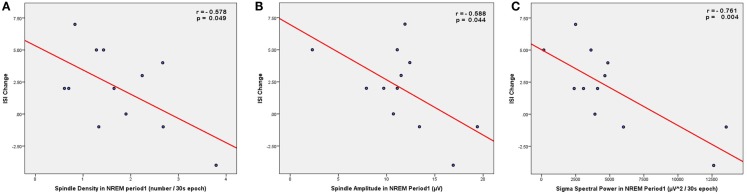
**Scatter plots showing the correlations between spindle parameters (A, density; B, amplitude) or sigma power (C) during NREM sleep period 1 (from C4–O2 EEG derivation) and the change in insomnia severity index (ISI) from the low stress to the high stress period**.

Given that PSQI did not show significant increase in response to academic stress, bivariate correlations were performed between spindle parameters or sigma power and ISI change only (Table 2). When examining spindle density, there was a significant negative correlation between spindle density during the first NREM sleep period and ISI change (Figure 3A), i.e., lower spindle density at the beginning of the night was associated with higher increases in sleep complaints in response to academic stress. The correlation was not significant for spindle density during the whole night or during any other NREM sleep period. When looking at the other spindle parameters, duration, and frequency did not correlate with ISI change. Spindle amplitude, however, negatively correlated with ISI change, when considering either the first (Figure 3B) or the third NREM sleep period. Finally, there was a significant negative correlation between sigma EEG power, for the whole night as well as for each NREM sleep period, and ISI change. This correlation with sigma power was the most significant during the first NREM sleep period (Figure 3C). FIRST score at baseline was not correlated with ISI change (*r* = −0.10; *p* = 0.75).

**Table 2:** **Correlations between baseline spindle parameters or sigma power and change in insomnia severity index from low to high stress period**.

Parameters	ΔISI	
	Pearson’s *r*	*p* value
Spindle density during total NREM sleep	−0.322	0.308
Spindle density in NREM period 1	−0.578	0.049*
Spindle density in NREM period 2	−0.156	0.628
Spindle density in NREM period 3	−0.378	0.226
Spindle density in NREM period 4	−0.192	0.549
Spindle amplitude during total NREM sleep	−0.502	0.097
Spindle amplitude in NREM period 1	−0.588	0.044*
Spindle amplitude in NREM period 2	−0.486	0.109
Spindle amplitude in NREM period 3	−0.591	0.043*
Spindle amplitude in NREM period 4	−0.545	0.067
Spindle duration during total NREM sleep	0.104	0.748
Spindle duration in NREM period 1	0.010	0.975
Spindle duration in NREM period 2	0.136	0.672
Spindle duration in NREM period 3	−0.074	0.819
Spindle duration in NREM period 4	0.164	0.610
Spindle frequency during total NREM sleep	−0.167	0.603
Spindle frequency in NREM period 1	−0.257	0.420
Spindle frequency in NREM period 2	−0.091	0.779
Spindle frequency in NREM period 3	−0.182	0.572
Spindle frequency in NREM period 4	−0.210	0.512
Sigma spectral power during total NREM sleep	−0.685	0.014*
Sigma spectral power in NREM period 1	−0.761	0.004**
Sigma spectral power in NREM period 2	−0.597	0.041*
Sigma spectral power in NREM period 3	−0.710	0.010**
Sigma spectral power in NREM period 4	−0.631	0.028*

*ΔISI, insomnia severity index change from low to high stress period; NREM period 1–4, first to fourth NREM sleep period*.

*Significance at *p* < 0.05.

**Significance at *p* < 0.01.

## Discussion

Taken together these results indicate that spindle activity constitutes a predisposing factor for the future aggravation of insomnia complaints in response to stress. They confirm our hypothesis that lower spindle density is associated with a higher vulnerability to sleep disturbances triggered by stress. In this study, the severity of insomnia symptoms assessed by ISI increased from the beginning to the end of the semester – a period coinciding with intense preparations for final examinations – thus validating our chosen model of stress-induced sleep disturbances. The validity of this academic stress model is further supported by the increase of self-reported perceived stress across the academic semester, as shown by the significant DASS-S increase with time. In addition, these results extended our initial hypothesis in two directions. First, we found that this predictive relationship between spindle density and ISI was restricted to the spindles during the first NREM sleep period, i.e., at the beginning of the night. Second, besides spindle density, we also observed significant correlations between ISI change and other spindle parameters, such as spindle amplitude and EEG power in the sigma frequency range, suggesting that spindle activity in general (and not only the mere presence of spindles) prospectively affects the evolution of insomnia symptoms.

The possible mechanisms underlying the relationship between spindle and stress-triggered insomnia complaints can be discussed in the light of previous studies investigating the functional properties of human sleep spindles. On the one hand, the filtering of external information at the thalamic level during sleep spindles might provide a potential mechanism for this predictive relationship. Tones presented during most of NREM sleep were found to activate the thalamus and the primary auditory cortex in an EEG/functional magnetic resonance imaging study; however, tones presented in coincidence with sleep spindles did not consistently activate thalamocortical auditory circuits (Dang-Vu et al., 2011). This result suggests that spindles provide a gating process to preserve the sleeping brain from disruption by sounds (and presumably also by other types of environmental stimulation). Based on this finding, a further study investigated the relationship between spindle density and the probability of maintaining sleep continuity under presentation of sounds with increasing intensities (Dang-Vu et al., 2010). At any sound intensity level, individuals with higher spindle density were more likely to preserve the continuity of sleep than subjects with lower spindle density. The ability of individuals with higher spindle density to more efficiently sleep throughout noise might provide them with a better capacity to resist sleep disturbances in response to stress. Exposure to acute stress is indeed known to enhance sensitivity to noise (Hasson et al., 2013), and is thus likely to increase vulnerability to sounds during sleep. Through the gating mechanisms associated with spindles, individuals with higher spindle density might be in a better position to counter the deleterious consequences of stress on noise sensitivity during sleep, leading to a lower propensity to insomnia complaints in response to stress. Future studies investigating sleep spindles in relation to acoustic stimulation in periods of high stress are needed to support this interpretation.

Spindles have also been shown associated with a variety of cognitive measures. Higher number of spindles and higher EEG sigma power have been shown positively correlated with better perceptual and analytical skills measured by the performance intellectual quotient (Fogel et al., 2007), as well as with higher score at the Raven progressive matrices test reflecting general cognitive abilities (Bodizs et al., 2005; Schabus et al., 2006). These findings suggest that spindles constitute a neurophysiological biomarker of intellectual capacities. Beyond these correlations with broad scores of cognitive abilities, previous studies also found that sleep spindle activity increased following procedural memory tasks such as motor sequence learning (Barakat et al., 2011) and mirror tracing task (Tamaki et al., 2008), as well as following declarative learning tasks (word pairs) (Gais et al., 2002; Schabus et al., 2004), and these increases correlated with overnight improvements of performance. Therefore, an alternative explanation for the relationship found in the present study is that individuals with higher spindle density or activity, given their higher cognitive abilities, might be more capable of efficiently learning their course materials and thus managing academic stress, which might ultimately make them less susceptible to sleep disturbances in such context. Both mechanisms – sleep-protective and cognitive – are not mutually exclusive and might act synergistically to confer individuals with higher spindle activity the ability to maintain sleep stability in the face of stress.

In this study, spindle density and other spindle parameters during stages N2–N3 were computed separately for each NREM sleep period, in addition to their quantification throughout the whole night. Indeed, spindle parameters do not remain constant throughout the night (De Gennaro et al., 2000). We observed a significant increase of spindle density and spindle duration over the course of the night (particularly between NREM sleep period 1 and each of the following NREM sleep periods). These observations are in line with previous studies showing increases of spindle density and duration across successive NREM sleep periods (De Gennaro et al., 2000; Martin et al., 2013). Interestingly, the modulation of spindle parameters throughout the night had an impact on the predictive relationships with sleep complaints, since spindle density only in the first NREM sleep period was significantly correlated with ISI change. This result suggests that sleep spindles during the early phase of the night have a predominant influence on the perception of changes in insomnia complaints. The reasons for such effects of spindles during early night remain unclear. Interestingly, a differential effect of spindle activity as a function of NREM sleep period was also observed in a study comparing spindle density between teenagers with major depressive disorder, teenagers at risk for depression and healthy controls (Lopez et al., 2010). In contrast to our results, depressed and at-risk teenagers had lower spindle density than controls during the third and fourth NREM sleep period, but not at the beginning of the night. These various results suggest a differential clinical significance for spindle activity depending on the corresponding NREM sleep period.

Our findings also demonstrated that, besides spindle density, other parameters reflecting spindle activity (spindle amplitude, sigma power) correlated with change of sleep quality in response to stress. The correlations with spindle amplitude and EEG sigma power suggest that spindle activity (i.e., intensity) – reflecting the degree of thalamocortical synchronization – also modulates the perception of stress-induced sleep disturbances. Other studies investigating the functional properties of sleep spindles also resorted to measures of spindle intensity: EEG sigma power correlated with scores of intellectual abilities (Fogel et al., 2007), and spindle amplitude modulated the reactivation during sleep spindles of brain regions involved in the encoding of a declarative learning task (Bergmann et al., 2012). In our study, correlation between sigma power and stress-induced sleep complaints was significant for all NREM sleep periods, while it was limited to the first NREM sleep period for spindle density (as well as the third for spindle amplitude). Such discrepancies between the effects of spindle density compared to those of sigma power were also observed in previous studies (Gais et al., 2002), which further underlines that EEG spectral power in the sigma frequency range cannot be fully equated to the detection of sleep spindles as discrete events. Indeed, sigma power also captures activities that do not meet the standard criteria for full-blown sleep spindles, and thus constitutes a more sensitive (but less specific) method for spindle quantification. Taken together these effects of spindle density, amplitude, and sigma power suggest that, although the contribution of spindles during early night seems more predominant, spindle activity throughout the whole night affects the perception of stress-induced changes in sleep quality.

If lower spindle density (and activity) constitutes a predisposing factor for the surge of insomnia complaints, it could be expected that chronic insomniacs would tend to demonstrate lower spindle measures compared to good sleepers. A previous study analyzed the differences in spindle density between chronic primary insomniacs and good sleepers, by performing a visual detection of sleep spindles on C3 derivation (Bastien et al., 2009). Surprisingly, no significant difference in spindle density was found between groups. Further studies are needed to replicate this result, and to extend it to other (more sensitive) modalities of spindle detection as well as to other measures of spindle activity such as spindle amplitude and sigma power. If confirmed, the absence of change in spindle activity in chronic insomniacs as a group might reflect the large heterogeneity in the clinical presentation and sleep characteristics even within primary insomniacs. For instance, the presence of objective sleep disturbances, as defined by PSG decreases in total sleep time and sleep efficiency, is not observed consistently across chronic insomniacs (Vgontzas et al., 1994). However, the presence of objective short sleep duration defines a subgroup of insomniacs with a distinct clinical profile exemplified by a higher risk for hypertension, diabetes, cognitive impairment, and mortality (Vgontzas et al., 2013). Likewise, it is possible that there is a subgroup of insomniacs characterized by a higher vulnerability to environmental disturbances due to a lesser amount of sleep-protective factors such as sleep spindles. In contrast, another subgroup might instead be characterized by less objective sleep disruption and a larger contribution of cognitive-emotional factors such as dysfunctional beliefs about sleep and higher levels of anxiety and worry. Considering the chronic insomnia population as a single group might dilute the alterations of sleep microarchitecture that possibly affect a subpopulation of insomniacs only. Future studies should further explore the quantification of sleep-protective mechanisms in chronic insomniacs and subgroups of insomniacs.

While the current study was primarily focused on the neurophysiological predictors of sleep disturbances through the assessment of spindle activity, it should be reminded that psychological and medical factors also play an important role in the incidence of insomnia complaints. For instance, mental health problems, maladaptive personality traits, a positive family history of insomnia, and an objectively shorter sleep duration on PSG were associated with a higher risk of evolution of poor sleep toward chronic insomnia (Fernandez-Mendoza et al., 2012). As for insomnia complaints in response to stress, specific questionnaires of vulnerability to stress-induced insomnia have been developed, such as the FIRST (Drake et al., 2004). Surprisingly, the FIRST score did not predict changes in ISI from low to high stress periods in our analysis. This might be explained by the high correlation between the FIRST score and ISI at baseline in this sample (*r*_FIRST-ISI_ = 0.86), leaving little room to predict change over time. Nevertheless, it has been previously shown that individuals with higher score on the FIRST (Drake et al., 2004) are more vulnerable to the first night effect (i.e., worse sleep quality during the first night of sleep recording in lab) and to the sleep-disrupting effects of caffeine (Drake et al., 2004, 2006), and demonstrate higher risk of developing persistent insomnia over time (Drake et al., 2014; Jarrin et al., 2014). Interestingly, the differential vulnerability to stress-induced insomnia may emerge from differences in hyperarousal predisposition, given the association between FIRST scores and indices of cognitive-emotional hyperarousal (Fernandez-Mendoza et al., 2010), which has recently been demonstrated to be (at least partially) heritable (Fernandez-Mendoza et al., 2014). Because sleep spindles modulate sleep stability in response to environmental stimulation (Dang-Vu et al., 2010, 2011), lower spindle activity – which has been found in the present study to predispose to higher increase of sleep disturbances – might be considered as a trait predisposing to a state of neurobiological hyperarousal in which individuals are more vulnerable to externally driven sleep disruption. Therefore, these various findings on the vulnerability to stress-induced insomnia can be integrated within the framework of the hyperarousal model for insomnia viewed from a psychophysiological perspective (Riemann et al., 2010).

There are several limitations to the current study. First, larger samples are needed to confirm these findings. Due to the limited number of participants, correction for multiple comparisons was not applied in the present data set, and thus our findings need replication. Second, only undergraduate university students were included in the present study due to the need of a naturally occurring stressor encompassing well-defined periods of lower and higher stress, as provided by the model of academic stress. Future studies should extend these findings to other populations and other types of stressors, including chronic stressors that may impact the persistence of insomnia complaints over time. Third, assessment of sleep quality and insomnia complaints was evaluated through self-reported questionnaires only: ISI and PSQI. The absence of significant PSQI change across the semester in our study might indicate that the impact of academic stress on sleep predominantly affects insomnia complaints rather than general sleep quality. Furthermore, the nature of the stressor (academic stress) precluded the repetition of objective sleep measurements with PSG, given the difficulty of having participants coming at the sleep laboratory during busy periods of final examinations. The use of more practical objective measures of sleep such as actigraphy measurements might constitute an interesting complement in future studies, in order to obtain not only objective but also prolonged assessments of sleep over several days or weeks. Finally, we restricted our analyses to spindle activity over central derivations (C4), given the centroparietal predominance of sleep spindle activity (De Gennaro et al., 2000). In order to avoid additional comparisons in our limited sample, distinction between fast and slow spindles was not performed in this present analysis. Our results, however, suggest that the frequency of spindles did not affect the change in insomnia symptoms, given the absence of correlation between spindle frequency and ISI change (Table 2). Future studies on larger samples could further evaluate the role of spindle frequency on sleep quality changes by analyzing the role of slow and fast spindles separately. The study of other EEG oscillations during sleep might also be of interest given previous results indicating the contribution of brain oscillations in other frequency bands, such as alpha rhythms (McKinney et al., 2011) and slow wave activity (Dang-Vu et al., 2011; Schabus et al., 2012), to the preservation of sleep continuity in the face of external stimulation.

## Conclusion

Our study provides the first evidence for the contribution of sleep neurophysiological activity to the prospective increase of sleep disturbances in response to a standardized stressor in a sample of young healthy volunteers. In line with previous findings indicating that sleep spindle constitutes a biomarker of sleep stability, our results suggest that spindle activity also represents a predisposing factor modulating the vulnerability to sleep disruption in conditions of stress. These results have implications for the understanding of the neural mechanisms underlying the evolution of sleep disturbances and particularly insomnia. They might also have clinical implications, by providing a biomarker for the identification of individuals at risk for future sleep disruption. Finally, our findings emphasize the potential importance of future therapeutic interventions aimed at enhancing sleep spindle activity in order to preserve sleep quality.

## Author Contributions

TDV and JPG designed the study. AS, SB, KW, and JOB acquired the data. TDV, AS, SB, MB, CB, and JG analyzed the data. TDV, AS, SB, and JPG interpreted the results. TDV wrote the manuscript. AS prepared the tables and figures. All the authors revised and commented the manuscript, gave their final approval of the manuscript, and agree to be accountable for all aspects of the work.

## Conflict of Interest Statement

The authors declare that the research was conducted in the absence of any commercial or financial relationships that could be construed as a potential conflict of interest.

## Supplementary Material

The Supplementary Material for this article can be found online at http://www.frontiersin.org/Journal/10.3389/fnhum.2015.00068/abstract
