# Supplementary material for: Sleep Spindles Predict Stress-Related Increases in Sleep Disturbances
Source: Front Hum Neurosci. 2015 Feb 10;9:68. doi: 10.3389/fnhum.2015.00068 (PMC4322643; doi:10.3389/fnhum.2015.00068)
Supplement: Supplementary file 1 [file Table_1.DOCX]

***Supplementary Material***

**Sleep spindles predict stress-related increases in sleep disturbances**

Thien Thanh DANG-VU^1,2,3,4,5,6*^, Ali SALIMI^1,2,3,4^, Soufiane BOUCETTA^1,2,3,4^, Kerstin WENZEL^6^, Jordan O’BYRNE^1,2,3,4^, Marie BRANDEWINDER^7^, Christian BERTHOMIER^7^ and Jean-Philippe GOUIN^3,5,6^

^1^Department of Exercise Science, Concordia University, Montréal, QC, Canada

^2^Center for Studies in Behavioral Neurobiology, Concordia University, Montréal, QC, Canada

^3^PERFORM Center, Concordia University, Montréal, QC, Canada

^4^Centre de Recherche de l’Institut Universitaire de Gériatrie de Montréal, Montréal, QC, Canada

^5^Center for Clinical Research in Health, Concordia University, Montréal, QC, Canada

^6^Department of Psychology, Concordia University, Montréal, QC, Canada

^7^Physip SA, Paris, France

Corresponding author:

* Dr Thien Thanh DANG-VU, MD PhD

Concordia University

Department of Exercise Science

Center for Studies in Behavioral Neurobiology and PERFORM Center

7141 Sherbrooke Street West, SP 165-27

Montreal, QC, H4B 1R6, Canada

Email: [tt.dangvu@concordia.ca](mailto:jp.gouin@concordia.ca)

**Supplementary Data**

Supplementary analysis were made in order to evaluate sigma power and spindle parameters exclusively during stage N2 NREM sleep periods. Repeated measure ANOVA tests showed a significant change in spindle duration across stage N2 NREM sleep periods (F=4.2, p=0.041). Additional Bonferroni Post-hoc tests demonstrated that spindle duration throughout the first period of stage N2 NREM sleep was significantly lower than during each of the three subsequent stage N2 NREM periods (p=0.019, 0.039, 0.048, respectively) (supplementary figure 1.C). There was a trend for an increase in N2 spindle density across sleep cycles, with only marginal significance however (p=0.079) (supplemental figure 1.A). No change in spindle amplitude (p=0.282), frequency (p=0.308), and sigma power (p=0.261) was observed across stage N2 NREM sleep periods (supplementary figure 1).

Bivariate correlations were performed between N2 spindles variables or sigma power and ISI change (supplementary table 1). Pearson correlation tests showed a significant negative correlation between ISI change and spindle amplitude during the first (supplementary figure 2.A) and the fourth period of stage N2 NREM sleep. In addition, sigma spectral power was negatively associated to ISI change for the whole night as well as during all the stage N2 NREM sleep periods. This negative correlation was most significant during the first period (supplementary figure 2.B).

1. **Supplementary Figures and Tables**

## Supplementary Tables

| **Supplementary Table 1. Correlations between baseline spindle parameters or sigma power and change in insomnia severity index from low to high stress period.** | | |
| --- | --- | --- |
|  | **Δ ISI** | |
| **Parameters** | **Pearson's *r*** | ***p* value** |
| Spindle density during total stage N2 | -0.157 | 0.626 |
| Spindle density in stage N2 Period 1 | -0.221 | 0.490 |
| Spindle density in stage N2 Period 2 | -0.010 | 0.975 |
| Spindle density in stage N2 Period 3 | -0.249 | 0.434 |
| Spindle density in stage N2 Period 4 | -0.098 | 0.762 |
|  |  |  |
| Spindle amplitude during total stage N2 | -0.485 | 0.110 |
| Spindle amplitude in stage N2 Period 1 | -0.675 | 0.016* |
| Spindle amplitude in stage N2 Period 2 | -0.545 | 0.067 |
| Spindle amplitude in stage N2 Period 3 | -0.567 | 0.054 |
| Spindle amplitude in stage N2 Period 4 | -0.579 | 0.048* |
|  |  |  |
| Spindle duration during total stage N2 | 0.249 | 0.434 |
| Spindle duration in stage N2 Period 1 | 0.086 | 0.791 |
| Spindle duration in stage N2 Period 2 | -0.080 | 0.804 |
| Spindle duration in stage N2 Period 3 | 0.022 | 0.946 |
| Spindle duration in stage N2 Period 4 | 0.026 | 0.936 |
|  |  |  |
| Spindle frequency during total stage N2 | -0.177 | 0.582 |
| Spindle frequency in stage N2 Period 1 | -0.379 | 0.225 |
| Spindle frequency in stage N2 Period 2 | -0.161 | 0.617 |
| Spindle frequency in stage N2 Period 3 | -0.332 | 0.491 |
| Spindle frequency in stage N2 Period 4 | -0.210 | 0.512 |
|  |  |  |
| Sigma spectral power during total stage N2 | -0.682 | 0.015* |
| Sigma spectral power in stage N2 Period 1 | -0.751 | 0.005** |
| Sigma spectral power in stage N2 Period 2 | -0.640 | 0.025* |
| Sigma spectral power in stage N2 Period 3 | -0.690 | 0.013* |
| Sigma spectral power in stage N2 Period 4 | -0.652 | 0.022* |
| *Δ ISI, Insomnia Severity Index change from low to high stress period; N2 Period 1-4, first to fourth stage N2 NREM sleep period; * significance at p < .05; ** significance at p < .01.* | | |

## Supplementary Figures

Supplementary Figure Legends

Supplementary Figure 1- Evolution of spindle parameters and sigma power across the four stage N2 NREM sleep periods: A. Spindle density; B. Spindle maximum amplitude; C. Spindle duration; D. Spindle frequency; E. EEG spectral power in the sigma frequency band. All values were extracted from C4-O2 EEG derivation. The dots represent the mean value, and the bars show the standard error of the mean. There was a significant increase of spindle duration from the first period of stage N2 NREM sleep to the subsequent N2 NREM periods (one-way repeated measures ANOVA, p < 0.05), and a trend for an increase in spindle density (p < 0.079).

**Supplementary Figure 2-** Scatter plots showing the correlations between spindle amplitude or sigma power during stage N2 NREM sleep Period 1 (from C4-O2 EEG derivation) and the change in Insomnia Severity Index (ISI) from the low stress to the high stress period.
