# Supplementary figures and images for: Sleep Spindles Predict Stress-Related Increases in Sleep Disturbances
Source: Front Hum Neurosci. 2015 Feb 10;9:68. doi: 10.3389/fnhum.2015.00068 (PMC4322643; doi:10.3389/fnhum.2015.00068)

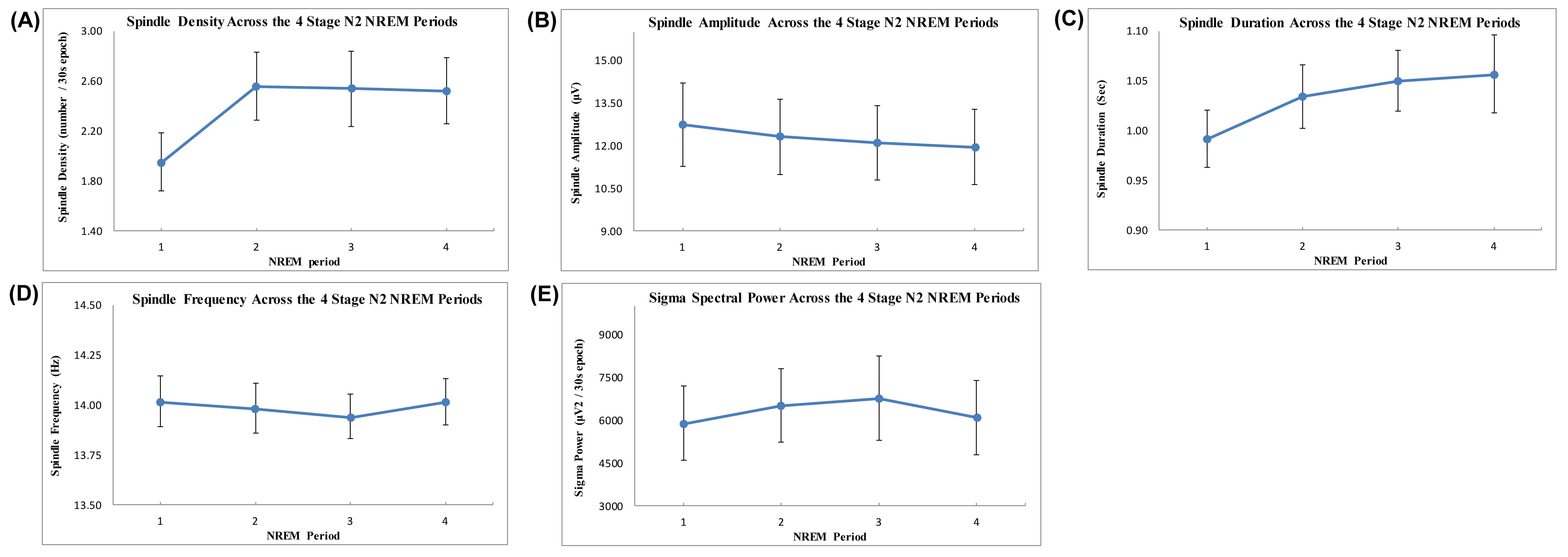

Supplement: Supplementary file 2 [file Image_1.JPEG]

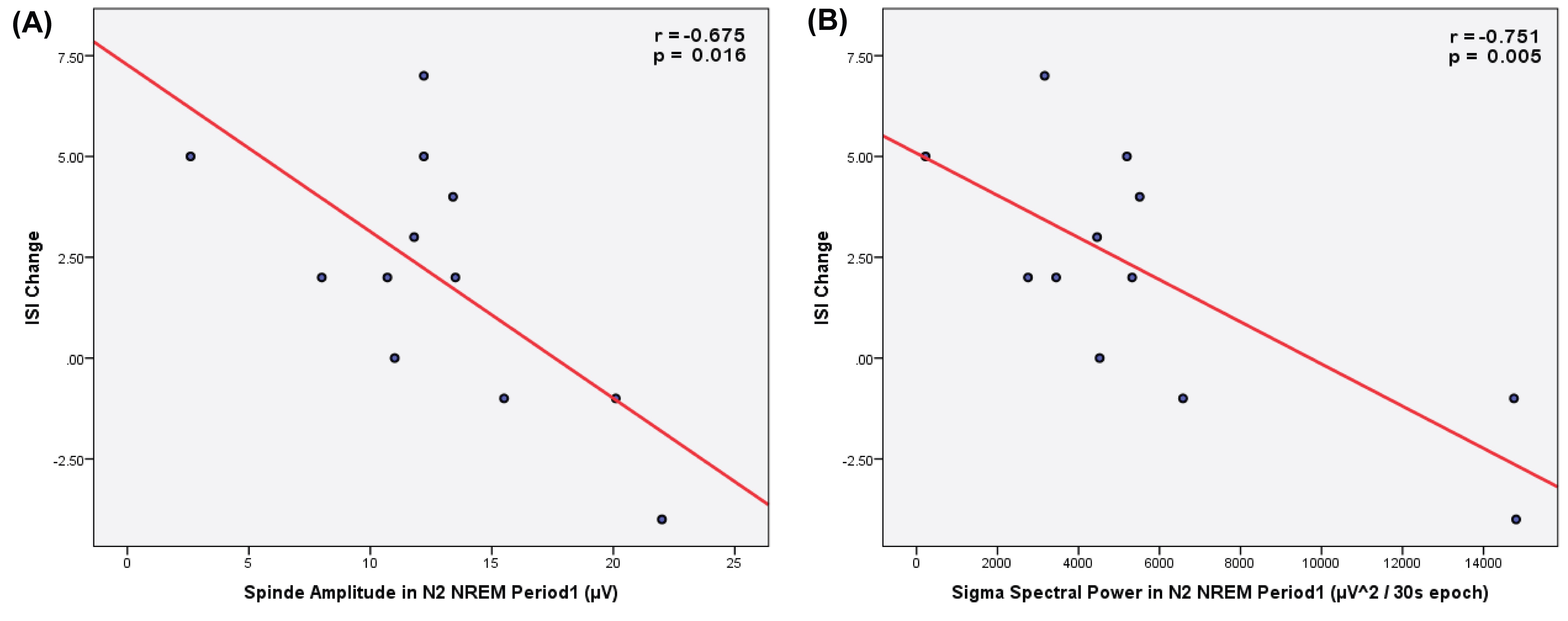

Supplement: Supplementary file 3 [file Image_2.JPEG]
